# The Heme-Based Oxygen-Sensor Phosphodiesterase *Ec* DOS (DosP): Structure-Function Relationships

**DOI:** 10.3390/bios3020211

**Published:** 2013-06-17

**Authors:** Toru Shimizu

**Affiliations:** 1 Department of Cell Biology, Shantou University Medical College, Shantou 515041, China; 2 Department of Biochemistry, Charles University in Prague, Prague 2, 128 43, Czech Republic; 3 Department of Medical Biotechnology, Jagiollonian University, Krakow 30-387, Poland; 4 Research Center for Compact Chemical System, National Institute of Advanced Industrial Science and Technology (AIST), Sendai 983-8551, Japan; E-Mail: shimizu@tagen.tohoku.ac.jp; Tel./Fax: +81-22-378-3985

**Keywords:** heme protein, oxygen sensor, phosphodiesterase, c-AMP, c-di-GMP, signal transduction

## Abstract

*Escherichia coli* Direct Oxygen Sensor (*Ec* DOS, also known as *Ec* DosP) is a heme-based O_2_-sensing phosphodiesterase from *Escherichia coli* that catalyzes the conversion of cyclic-di-GMP to linear di-GMP. Cyclic-di-GMP is an important second messenger in bacteria, highlighting the importance of understanding structure-function relationships of *Ec* DOS. *Ec* DOS is composed of an N-terminal heme-bound O_2_-sensing PAS domain and a C-terminal phosphodiesterase catalytic domain. Notably, its activity is markedly enhanced by O_2_ binding to the heme Fe(II) complex in the PAS sensor domain. X-ray crystal structures and spectroscopic and catalytic characterization of the wild-type and mutant proteins have provided important structural and functional clues to understanding the molecular mechanism of intramolecular catalytic regulation by O_2_ binding. This review summarizes the intriguing findings that have obtained for *Ec* DOS.

## 1. Introduction

**Figure 1:**
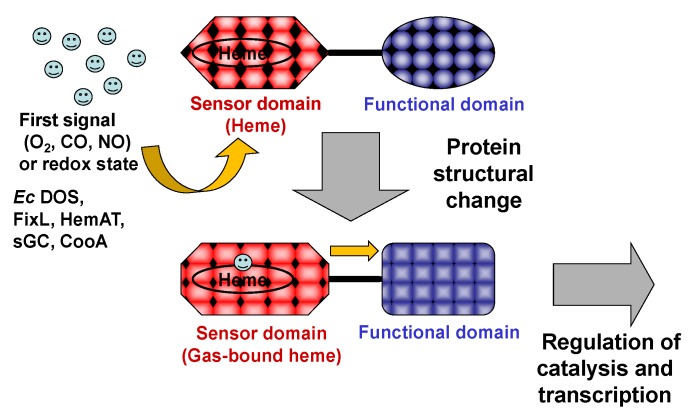
General concept of heme-based gas sensors with intramolecular signal transduction properties. The heme iron complex is bound to the sensor domain located in the N-terminus; the functional domain is located in the C-terminus. The gas molecule (O_2_, NO, or CO) is the first signal (upper). Association of the first signal with the heme Fe(II) complex (or dissociation from it), changes the protein structure (lower). This protein structural change constitutes the second signal, which is transduced to the functional domain, switching on (or off) functions such as phosphodiesterase (PDE), diguanylate cyclase (DGC), histidine kinase (HK), or transcription. Adapted from [7].

Heme proteins play important roles in O_2_ storage (myoglobin [Mb]), O_2_ transfer (hemoglobin [Hb]), O_2_ activation (cytochrome P450, nitric oxide synthase), electron transfer (cytochromes), and many more functions. In addition to these well-known prototypical heme proteins, is an emerging class of heme-based gas-sensing proteins [1,2,3,4,5,6]. In general, the heme-based gas-sensing protein is composed of two domains: an N-terminal sensor domain and a C-terminal functional domain (Figure 1). The heme iron complex bound to the sensor domain acts as the gas sensor. Association of the gas molecule to the heme iron complex or dissociation from it alters the protein structure in the heme-bound sensor domain. In this process, binding of the gas molecule is the first signal and the protein structural alteration near the heme-bound site becomes the second signal. This second signal is then transduced to the C-terminal functional domain, switching on (or off) the associated activity, which may consist of phosphodiesterase (PDE), diguanylate cyclase (DGC), histidine kinase (HK), or transcription, among others. A number of representative gas-sensing proteins, including heme-based oxygen sensors such as the *Escherichia coli* Direct Oxygen Sensor (*Ec* DOS, also known as *Ec* DosP), NO sensors such as soluble guanylate cyclase (sGC), and CO sensors such as CooA, have been reported [1,2,3,4,5,6].

The O_2_ molecule is among the most abundant gas molecules in the environment and is important for numerous physiological functions; thus, O_2_-sensor proteins are physiologically important in living organisms. The properties of O_2_ are much different from those of other gaseous molecules such as NO and CO, which are also important in various physiological and pathological processes. Prokaryotes possess well-characterized heme-based oxygen-sensor enzymes such as *Ec* DOS, FixL, and HemAT. The protein fold of the heme-binding domain of *Ec* DOS and FixL is the PAS (Per-Arnt-Sim) fold, whereas that of HemAT is the globin fold. *Ec* DOS is a phosphodiesterase (PDE), whereas FixL is a histidine kinase (HK). A precise, concrete catalytic function has not yet been assigned to HemAT, although it is certain that HemAT is involved in methylchemostasis, thus sensing O_2_ concentration and allowing cells to move toward the environment with a higher O_2_ concentration. Accordingly, the molecular O_2_-sensing and intramolecular signal transduction mechanisms of these heme-based oxygen sensors should be significantly different from each other [1,2,3,4,5,6]. 

The PAS fold, comprised of approximately 100 to 120 amino acids, is characterized by several α-helices flanking a five-stranded antiparallel β-sheet scaffold. PAS domains occur in proteins from all kingdoms of life. PAS domains are important signaling modules in that PAS ligand binding either functions as a primary cue to initiate a cellular signaling response, or provides the domain with the capacity to respond to first physical or chemical signals such as gas molecules, redox potential, or photons [8,9,10]. 

The substrate of *Ec* DOS is c-di-GMP, which is an important second messenger in bacteria; thus, *Ec* DOS is predicted to be critically involved in numerous physiological functions of *E. coli*. The association of *Ec* DOS with YddV (or *Ec* DosC), a heme-based oxygen sensor DGC, in a larger protein complex is known to be involved in intricate novel regulations including the RNA regulation, which should be linked to oxygen status through the *Ec* DOS/YddV complex [11,12]. Perhaps, not surprisingly, given the crucial role of *Ec* DOS in *E. coli* physiology and interest in its intramolecular signal transduction mechanism, *Ec* DOS structure-function relationships have been studied intensively. This research interest also stems, in part, from the fact that efficient protein overexpression and purification methods for *Ec* DOS have been well characterized [13,14]. The present review focuses on *Ec* DOS, summarizing and discussing catalytic, structural, physicochemical, and genetic findings, and application of a previously reported protein microarray system to understanding the function of this interesting enzyme.

## 2. Catalytic Activities

**Figure 2:**
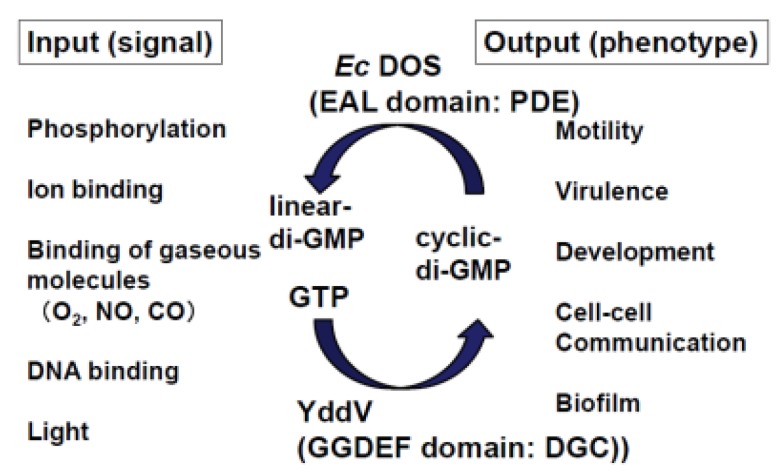
Input signals and output phenotypes of c-di-GMP metabolism. C-di-GMP is an important second messenger for numerous bacterial functions [15,16,17,18]. Various input signals (first signals) regulate degradation or synthesis of c-di-GMP via PDE or DGC activity, respectively, manifesting as different physiological functions. In *E. coli*, the PDE activity toward c-di-GMP is exerted by the EAL domain of *Ec* DOS, whereas the DGC activity toward c-di-GMP is exerted by the GGDEF domain of YddV. Adapted from [18].

**Figure 3:**
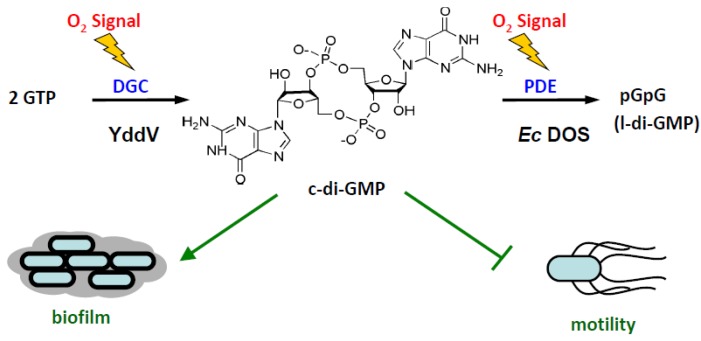
Bacterial biofilm is formed by c-di-GMP; thus, c-di-GMP inhibits bacterial motility. For example, in *E. coli*, both *Ec* DOS (PDE activity) and YddV (DGC activity) are heme-based oxygen-sensor enzymes; thus, their catalytic activities are switched on by binding of the first signal, O_2_, to the heme Fe(II) complex. Note that O_2_ affinity to YddV is more than five-fold higher than that of *Ec* DOS, thus YddV is more sensitive to O_2_ than *Ec* DOS.

c-di-GMP is an important second messenger involved in bacterial motility, virulence, development, cell-cell communication, biofilm formation, and numerous other functions (Figure 2) [15,16,17,18]. The C-terminal functional domain of *Ec* DOS has both EAL and GGDEF subdomains, which are normally associated with c-di-GMP-linearizing (PDE) and c-di-GMP-synthesizing (DGC) activity, respectively [15,16,17]. However, accumulating experimental evidence suggests that *Ec* DOS acts as c-di-GMP-specific PDE, but does not exhibit DGC activity. Instead, YddV, a heme-based oxygen sensor, has been found to function as a DGC in *E. coli* [11,12,19]. Thus, two heme-based oxygen sensors, *Ec* DOS and YddV, function synergistically to regulate c-di-GMP concentration in *E. coli* in response to various stimuli, leading, for example, to sticky biofilm formation (high c-di-GMP) or high mobility (low c-di-GMP) (Figure 3). 

**Figure 4:**
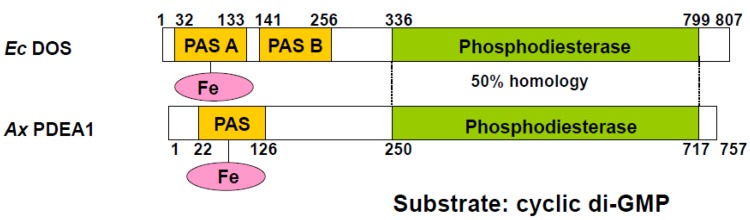
Both *Ec* DOS and *Ax*PDEA1 are heme-based oxygen-sensor PDEs toward c-di-GMP. The EAL domain associated with PDE is located in the C-terminal domain. The activity of *Ec* DOS is enhanced by O_2_ binding to the heme Fe(II) complex, whereas that of *Ax*PDEA1 is enhanced by O_2_ dissociation from the heme Fe(II) complex [11,12,20,23,24,25,26].

On the basis of the amino acid sequence of *Ec* DOS, it was predicted that the PDE activity of this enzyme toward c-di-GMP is regulated by O_2_ association with, or dissociation from, the N-terminal PAS domain-bound heme Fe(II) complex [20]. Physicochemical studies have been carried out using the isolated heme-bound PAS domain of *Ec* DOS (*Ec* DOS-PAS-A) [21]. The initial studies using c-AMP as a substrate characterized the catalytic activities of *Ec* DOS because of difficulties in obtaining sufficient quantities of high-quality c-di-GMP (see below) [22]. In contrast, the PDE catalytic activity of AxPEDA1, a heme-based oxygen-sensor PDE from *Acetobacter xylinum*, toward c-di-GMP has been more directly characterized. These studies have shown that the activity of this enzyme is stimulated by O_2_ dissociation from the heme Fe(II) complex (Figure 4) [23].

### 2.1. Catalytic Activity toward c-AMP

#### 2.1.1. Heme Redox-Dependent PDE Activity toward c-di-AMP

**Figure 5:**
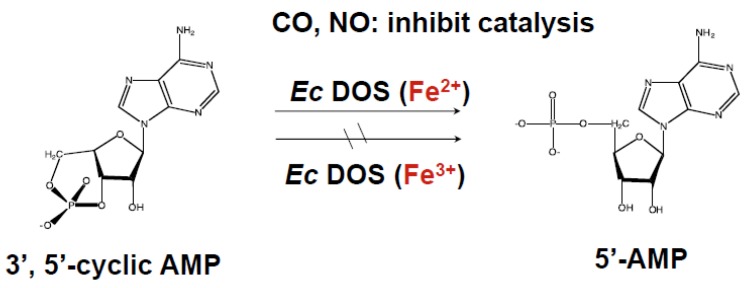
Catalysis of *Ec* DOS heme Fe(II) complex toward c-AMP is much higher than that for *Ec* DOS heme Fe(III) complex [22]. Addition of NO or CO to the heme Fe(II) complex-containing form inhibits catalysis.

**Figure 6:**
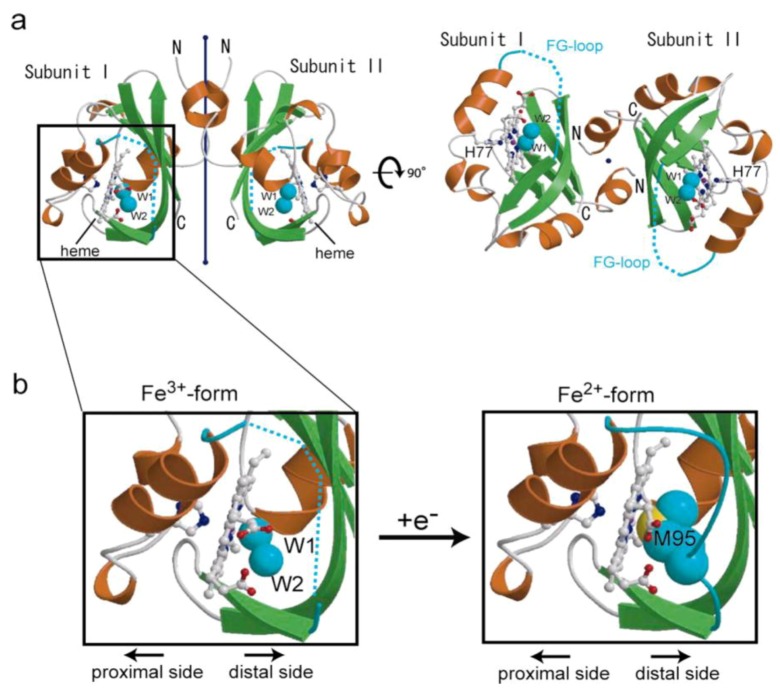
Heme redox-dependent structural changes in *Ec* DOS-PAS-A (PDB codes: 1V9Y and 1V9Z) [27,28]. The protein structure of the heme Fe(III) complex, a catalytically inactive form toward c-AMP, is flexible; thus the F-G loop cannot be determined, and the 6th axial ligand appears to be the hydroxide anion. In contrast, the protein structure of the heme Fe(II) complex, a catalytically active form toward c-AMP, is rigid, enabling determination of the F-G loop and identification of M95 as the 6th axial ligand. Adapted from [27].

The PDE catalytic activity of *Ec* DOS toward c-AMP is much lower than that toward c-di-GMP. Nevertheless, interesting findings have been obtained for *Ec* DOS using c-AMP as a substrate. Specifically, significant PDE activity toward c-AMP was observed for the full-length *Ec* DOS containing the heme Fe(II) complex (*Ec* DOS-heme Fe(II)), whereas activity was negligible for the full-length *Ec* DOS containing the heme Fe(III) complex (*Ec* DOS-heme Fe(III)) (Figure 5) [22], demonstrating that the heme iron redox state of *Ec* DOS regulates catalysis. This heme iron redox-dependent catalytic difference could be rationally explained by invoking heme redox-dependent protein structural changes [27]. Consistent with this, the crystal structure of *Ec* DOS-PAS-A in its inactive heme Fe(III) complex form (*Ec* DOS-PAS-A-heme Fe(III)) revealed a flexible protein; thus, a portion of the peptide in the heme surroundings, termed the F-G loop, and the structure of the heme distal side could not be determined. In contrast, *Ec* DOS-PAS-A in its active heme Fe(II) complex form (*Ec* DOS-PAS-A-heme Fe(II)) was shown to possess a rigid protein structure, enabling the structure of the heme distal side to be determined (Figure 6) [27,28]. These oxidation status-dependent differences in protein structure and PDE activity imply that *Ec* DOS is a redox-sensing PDE toward the substrate, c-AMP [29]. Note that heme-regulated catalytic regulation toward c-AMP is different from that toward c-di-GMP, as shown below.

Interestingly, the PDE catalysis of c-AMP by *Ec* DOS-heme Fe(II) is markedly suppressed by the binding of NO or CO [22], suggesting that binding of these gas molecules to the heme Fe(II) complex changes the protein structure of the heme environment, leading to suppression of catalysis. These findings further support the idea that *Ec* DOS is a gas-sensing enzyme. However, it has not been feasible to examine the effect of O_2_ binding to the heme Fe(II) complex on c-AMP catalysis, because the heme Fe(II)-O_2_ complex is not stable for the length of time required to measure the catalytic activity of this complex (>1 h).

#### 2.1.2. Removal of Heme or Truncation of the Heme-Bound PAS Domain from *Ec* DOS Does Not Influence the Catalytic Activity toward c-AMP

Since the heme redox state and the binding of NO or CO substantially alter catalytic activity toward c-AMP, it was thought that the heme iron complex or the heme-bound PAS domain would be essential for this activity. Unexpectedly, however, the catalysis of c-AMP by *Ec* DOS was not changed by removing the heme, or truncating the heme-bound PAS domain [21]. This suggests that attachment of the heme iron complex or the heme-bound PAS domain to the catalytic domain serves to inhibit catalysis, but is not essential for catalytic activity toward c-AMP. This observation has also been corroborated for PDE catalytic activity toward c-di-GMP, as noted below.

#### 2.1.3. Addition of *Ec* DOS-PAS-A to the Full-Length *Ec* DOS Enzyme Regulates Catalysis

It was thought possible that various types of small PAS proteins similar to the isolated PAS sensor domain are present in cells and could regulate the functions of other proteins in a manner similar to that of the calcium-binding protein, calmodulin, although no such proteins had been identified and/or purified. This theoretical possibility was therefore tested by examining the catalysis of full-length *Ec* DOS in the presence of *Ec* DOS-PAS-A [21]. 

**Figure 7:**
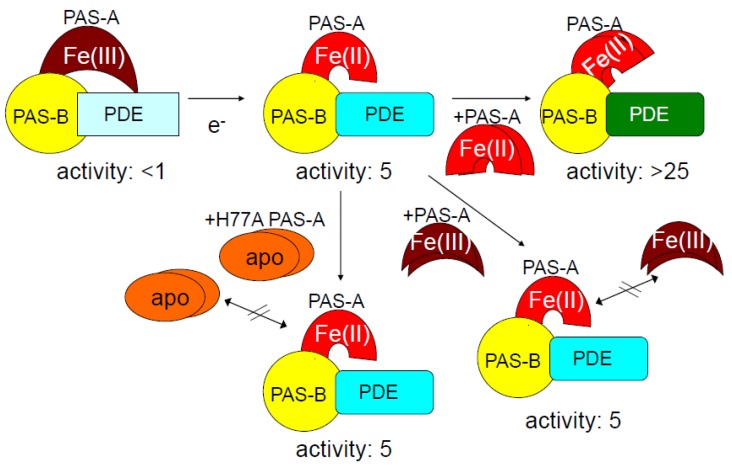
Addition of *Ec* DOS-PAS-A-heme Fe(II) to full-length *Ec* DOS-heme Fe(II) enhanced catalytic activity toward c-AMP by five-fold [21]. This catalytic enhancement was not observed upon adding *Ec* DOS-PAS-A-heme Fe(III) or heme-free (apo) *Ec* DOS-PAS-A to full-length *Ec* DOS-heme Fe(II). Furthermore, addition of *Ec* DOS-PAS-A-heme Fe(II) to PAS-A-truncated *Ec* DOS did not enhance catalytic activity. Therefore, the enhancement of catalytic activity is likely caused by protein–protein (PAS-A–PAS-A) interaction, as demonstrated by the novel protein microarray [30,31] (see Figure 10). Adapted from [21].

Interestingly, addition of *Ec* DOS-PAS-A containing the heme Fe(II) complex (*Ec* DOS-PAS-A-heme Fe(II)) to the full-length enzyme containing a heme Fe(II) complex (*Ec* DOS-heme Fe(II)) markedly enhanced catalysis (~five-fold), whereas addition of *Ec* DOS-PAS-A containing the heme Fe(III) complex (*Ec* DOS-PAS-A-heme Fe(III)) or heme-free (apo) *Ec* DOS-PAS-A to *Ec* DOS-heme Fe(II) did not change catalytic activity (Figure 7). In contrast, addition of *Ec* DOS-PAS-A-heme Fe(II) to an N-terminal PAS-A-truncated *Ec* DOS deletion mutant did not alter catalytic activity. Thus, the enhanced activity observed upon adding *Ec* DOS-PAS-A-heme Fe(II) to *Ec* DOS-heme Fe(II) likely reflects structural changes in the catalytic site resulting from interactions between *Ec* DOS-PAS-A and the PAS-A domain in the full-length *Ec* DOS. This protein–protein (PAS-A–PAS-A) interaction was demonstrated by sodium dodecyl sulfate-polyacrylamide gel electrophoresis (SDS-PAGE) as well as using a protein microarray system, as shown later. Although the interaction between the isolated PAS sensor domain and the full-length enzyme in this case was artificial, in general, a similar interaction should exist in nature and may be important for as yet unidentified catalytic regulation. 

#### 2.1.4. Issues of Catalysis toward c-AMP and Redox Sensor

It can be claimed that catalysis toward c-AMP is not assigned as a physiological function *in vivo* [20], since the turnover number, 0.15 min^−1^, of *Ec* DOS toward c-AMP [22] is significantly lower than that, 61 min^−1^, of CpdA, a prototype of c-AMP PDE in *E. coli* [32]. In addition, the turnover number of *Ec* DOS toward c-AMP is 300-fold lower that that toward c-di-GMP [11,12,24,25,26]. Furthermore, any reductants or reductase to regulate the heme redox state in *E. coli* has not been discovered. Reduction potential of *Ec* DOS around 45–70 mV *versus* standard hydrogen electrode (SHE) is similar to Mb, further implying that *Ec* DOS could not be called a physiologically relevant redox sensor. 

### 2.2. Catalysis of c-di-GMP

#### 2.2.1. O_2_ (NO/CO) Binding to the Heme Fe(II) Complex Enhances Catalysis via Dissociation of the Axial Ligand, M95

The catalytic domain of *Ec* DOS has both EAL and GGDEF sequences, which are expected to correspond to c-di-GMP linearization (PDE) and synthesis (DGC) functionalities, respectively (Figure 2, Figure 3, Figure 4) [5,11,12,16,17,18,20]. Accordingly, even though *Ec* DOS can clearly act on c-AMP, as evidenced by heme redox-regulated catalysis (Figure 5), the native substrate of *Ec* DOS is predicted to be c-di-GMP rather than c-AMP.

**Figure 8:**
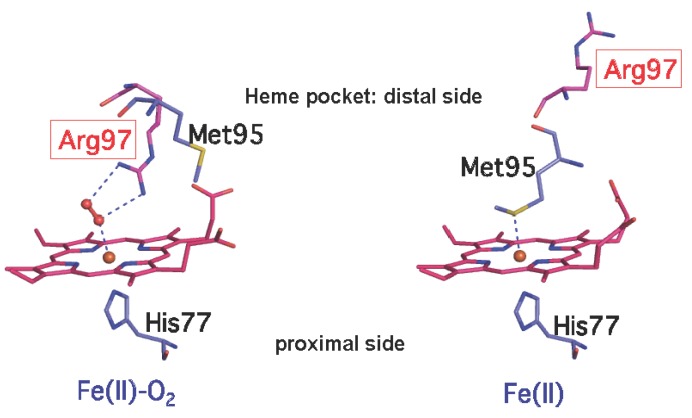
Structures of the heme pockets of the heme Fe(II)-O_2_ complex (**left**, PDB code: 1VB6) and the heme Fe(II) complex (**right**, PDB code: 1V9Z) of *Ec* DOS-PAS-A [27,28]. The catalytic enhancement of *Ec* DOS by added O_2_ (or NO/CO) is caused by dissociation of the 6th axial ligand M95 from the heme Fe(II) complex. Dissociation of M95 releases the locked state in the heme environment, allowing catalysis to occur. The O_2_ molecule is represented by the orange dumbbells. Adapted from [33].

It has been confirmed that *Ec* DOS has PDE activity toward c-di-GMP and its basal PDE activity toward c-di-GMP is substantially stimulated by binding of O_2_ (as well as NO and CO) to the heme Fe(II) complex in the enzyme [24,25]. Furthermore, M95, the axial ligand to the 6-coordinated heme Fe(II) complex in *Ec* DOS, was found to play a critical role in regulating the catalytic activity toward c-di-GMP. Specifically, from a coordination chemistry point of view, upon binding of the exogenous axial ligand O_2_ (or NO/CO) to the heme Fe(II) complex in the wild-type (WT) protein, the internal 6th axial ligand, M95, must first dissociate from the heme plane. In the next step, the exogenous axial ligand is bound to the heme Fe(II) complex [27,28]. Thus, it would appear that the protein movement caused by dissociation of M95 from the heme iron complex pushes part of the protein on the heme distal site upward or causes it to twist, leading to catalytic enhancement via intramolecular signal transduction. This supposition was tested by examining how mutations at M95 influence catalytic activity toward c-di-GMP. For M95A and M95L mutants, in which the heme iron complex takes the 5-coordinated form because there is no axial ligand at the 6th position, basal catalytic activities were high without the external axial ligand and further addition of the exogenous ligand, O_2_ (or NO/CO), did not significantly enhance catalysis. Interestingly, for M95H, where His is coordinated to the heme Fe(II) complex and a 6-coordinated complex is formed, addition of external ligands induced a catalytic enhancement similar to that observed for the WT enzyme. On the basis of these findings, it was proposed that M95 is coordinated to the heme Fe(II) complex under basal conditions and suppresses the catalysis. Subsequent binding of O_2_ (or NO/CO) to the heme Fe(II) complex unlocks the suppression and allows catalytic enhancement (Figure 8) [27,28].

#### 2.2.2. Addition of the Exogenous Ligands, Cyanide and Imidazole, to *Ec* DOS-Heme Fe(III) Stimulates Catalysis

We have studied the effects of the non-physiological exogenous axial ligands, cyanide, and imidazole, on the catalytic activity of *Ec* DOS-heme Fe(III). Surprisingly, the PDE catalytic activity toward c-di-GMP was substantially enhanced by adding these ligands [26]. Taken together with the results obtained for both heme Fe(II) and Fe(III) complex forms of *Ec* DOS, these results again suggest that an upward or twisting movement of part of the protein in the heme distal side of *Ec* DOS caused by exogenous ligands, a movement mimicked by mutations at M95, is an important factor in enhancing the catalytic activity toward c-di-GMP. 

#### 2.2.3. The Heme Iron Complex Is Not Essential for Intrinsic Catalytic Activity

Because binding of the exogenous axial ligand to both heme Fe(II) and Fe(III) complex forms substantially enhances catalysis of c-di-GMP, the effects of heme deletion from the PAS sensor domain of *Ec* DOS were examined. The heme-deleted mutant enzyme exhibited catalytic activity similar to that of the WT enzyme stimulated with exogenous axial ligands [26], an observation reminiscent of the activity toward c-AMP [21]. These results again suggest that the heme in the PAS sensor domain serves to suppress catalysis and that M95 acts to lock the catalytic activity of *Ec* DOS containing a heme Fe(II) complex. Binding of O_2_ (or NO/CO) to the heme Fe(II) complex releases the locked state, leading to enhanced catalytic activity (Figure 8). 

#### 2.2.4. Catalysis with Mn^2+^ Proceeds without Gas Molecules

**Figure 9:**
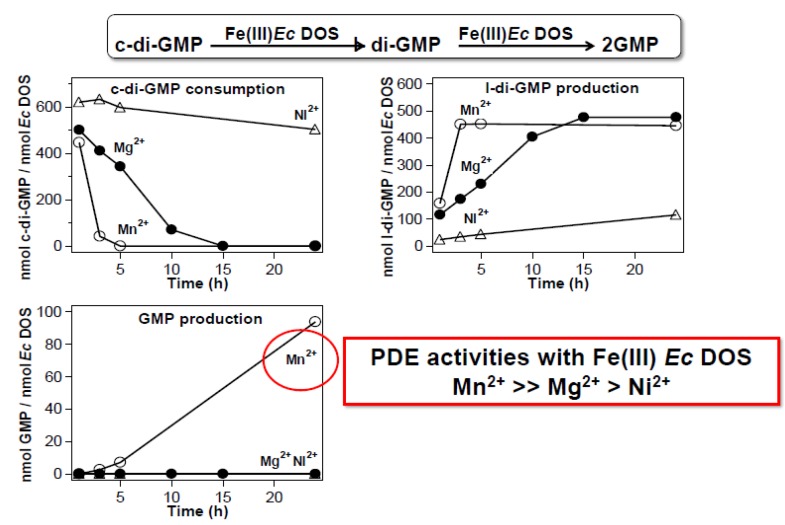
Mn^2+^ facilitates PDE catalytic activity toward c-di-GMP, but the catalytic mechanism is different from that of Mg^2+^. In the presence of Mn^2+^, full-length *Ec* DOS containing a heme Fe(III) complex exhibits fully competent PDE functions, catalyzing the two-step conversion of c-di-GMP all the way to GMP via l-di-GMP [35]. The first reaction from c-di-GMP to l-di-GMP is much faster with Mn^2+^ than with Mg^2+^, and the second reaction (cleaving l-di-GMP to form GMP) does not occur in the presence of Mg^2+^. Adapted from [35].

Mn^2+^, like Mg^2+^, mediates phosphorylation, dephosphorylation, and PDE reactions that facilitate catalytic activity [34]. To understand how Mn^2+^ behaves in the heme-based oxygen-sensor PDE, *Ec* DOS, the catalysis of c-di-GMP in the presence of Mn^2+^ instead of Mg^2+^ was examined [35]. The study showed that, in the presence of Mn^2+^, *Ec* DOS-heme Fe(III) mediates the two-step hydrolysis of c-di-GMP into GMP via the linear-di-GMP intermediate (Figure 9). By contrast, in the presence of Mg^2+^, the first linearization reaction of c-di-GMP is much slower than that in the presence of Mn^2+^, and the second reaction to form GMP is not observed for *Ec* DOS-heme Fe(III) [25,26]. Thus, *Ec* DOS-heme Fe(III), which is inactive in the presence of Mg^2+^, exhibits PDE activity in the presence of Mn^2+^, implying that the Mn^2+^ binds to the catalytic domain and that Mn^2+^-bound form of *Ec* DOS without exogenous ligands mimics the Mg^2+^-bound form, which is only induced upon O_2_ (or NO/CO) binding to *Ec* DOS-heme Fe(II). It has been speculated that Mn^2+^ may be situated or coordinated at an active-site position that, even in *Ec* DOS-heme Fe(III), is suitable for promoting optimal catalytic activity comparable to that observed for the heme Fe(II)-O_2_ form with Mg^2+^. Thus, one plausible explanation for the gas-sensing function of *Ec* DOS is that O_2_ (or NO/CO) binding enhances Mg^2+^ affinity for the active site and/or creates a Mg^2+^ coordination structure that is optimal for efficient PDE catalysis. 

#### 2.2.5. Interactions between Hydrogen Sulfide and the Wild Type and R97 Mutant Proteins

Hydrogen sulfide (H_2_S) has emerged as the fourth gaseous signaling molecule after O_2_, NO, and CO, and has been shown to play important roles in pathological and physiological functions [36,37,38]. The catalytic activity of *Ec* DOS-heme Fe(III) toward c-di-GMP was found to be substantially stimulated by added H_2_S [39]. It was reasoned that the active complex forms, heme Fe(III)-SH and heme Fe(II)-O_2_, generated by adding excess H_2_S, and modification of some C residues in the catalytic domain by added H_2_S significantly contributed to the catalytic enhancement by H_2_S. However, high physiologically irrelevant concentrations of H_2_S (>100 μM) were needed for catalytic stimulation. It is important to note that NO and CO are not physiological candidates for *Ec* DOS, although catalytic activation toward c-di-GMP was shown by those gas molecules [24,25]. Despite this lack of selectivity at the level of enzymatic activation, there are kinetic/themodynamic selectivities along with physiological availability, which makes possible to assign a native *versus* chemical signaling [3,4].

R97 is the direct O_2_-binding site in the heme Fe(II)-O_2_ complex of *Ec* DOS-PAS-A (Figure 8) [28]. Accordingly, the autoxidation rates of mutants at the R97 residue are very fast [33]. Upon addition of H_2_S to the R97A and R97I mutants of *Ec* DOS-PAS-A-heme Fe(III), an O_2_-incorporated modified heme is formed with absorption band characteristic of verdoheme [40]. Time-dependent mass spectroscopic analyses confirmed that this treatment resulted in the formation of verdoheme and a trace of biliverdin, suggesting that H_2_O_2_ or superoxide anion generated by these mutants would help to form verdoheme and biliverdin. 

## 3. Protein Structures

### 3.1. X-ray Crystal Structures: Ligand- and Redox-Dependent Conformational Changes

It has proved difficult to solve the crystal structure of full-length *Ec* DOS, but the X-ray crystal structures of *Ec* DOS-PAS-A have been determined [27,28]. These studies revealed substantial heme redox-dependent and O_2_-induced structural changes for *Ec* DOS-PAS-A [28]. Moreover, these changes in protein structure were highly concordant with exogenous ligand- and redox-dependent catalytic regulation [5,11,12,22,24,25,26,41,42]. 

The structure of the heme Fe(II)-O_2_ complex differs markedly from that of the heme Fe(II) complex, showing striking O_2_-dependent conformational changes in the F-G loop turn, the early G_β_-strand, and the HI turn (Figure 6, Figure 8) [28]. M95 located in the G_β_-strand is the axial ligand on the distal side of the heme Fe(II) complex of *Ec* DOS-PAS-A, whereas R97 located in the same strand is the direct O_2_-binding site in the heme Fe(II)-O_2_ complex (Figure 8) [28]. In the absence of O_2_ ligation, the R97 side chain is oriented toward the protein surface. O_2_ coordination is accompanied by a nearly 180° rotation of the side chain of R97. The side chain of M95 also adopts radically different orientations in the two ligation states such that it rotated 180° from the heme to point toward the protein surface (Figure 8). This motion involves a 10.91 Å change in the M95 sulfur atom. This striking ligand-dependent main chain distortion of M95 is transmitted further along the chain to the area between residues 89 and 96, resulting in a major distortion of the F-G loop turn. On the other hand, the ligand-dependent structural changes on the heme proximal side are less substantial. H77 was found to be the axial ligand on the proximal side for both O_2_-bound and -unbound complexes. The most significant structural difference between the complexes is the ~115° ring rotation of Y80 caused by O_2_ binding. The Arg residue also serves to stabilize the O_2_ molecule in the heme Fe(II)-O_2_ complex of FixL, a heme-based oxygen-sensor kinase that contains a PAS fold [43]. In *Ec* DOS, structural differences between the O_2_-bound and -unbound complexes include hydrogen networks comprising the heme propionates, N84 and R97.

*Ec* DOS-heme Fe(II) is active toward c-AMP, whereas *Ec* DOS-heme Fe(III) is inactive [5,21,22,41]. This redox-dependent catalytic difference is reflected in the structural differences between the different redox complexes (Figure 6) [27]. The protein structure of the inactive heme Fe(III) complex is relatively flexible, preventing determination of the protein structure on the heme distal side containing the F-G loop. In contrast, the active heme Fe(II) complex is sufficiently rigid to allow its protein structural features, including the F-G loop, to be determined. This difference in protein flexibility may be one of the key factors that determine catalytic activity toward c-AMP. Another interesting redox-dependent structural difference is that the axial ligand of the heme distal side of the heme Fe(III) complex is OH^−^, whereas that of the heme Fe(II) complex is M95, as described above. This marked redox-dependent ligand-switching in the heme distal side that accompanies the refolding of the F-G loop appears to be correlated with the ligand (O_2_)-dependent structural changes in the heme distal side, and results in the redox- and ligand-dependent catalytic regulation of *Ec* DOS. Redox-dependent rearrangement of the hydrogen bond network in the heme environment is also observed.

### 3.2. Domain Structures

The results of gel-exclusion chromatography suggest that full-length *Ec* DOS (amino acids 1–807) is a tetramer. This aspect of *Ec* DOS has been further examined by generating several truncated mutants and testing their catalytic activity, oligomer formation, and domain-domain interactions [21]. The heme-free mutant of full-length *Ec* DOS and heme-domain-truncated *Ec* DOS retained full catalytic activity toward c-AMP. This result supports the interpretation that the presence of the heme iron complex in the intact enzyme serves to suppress catalysis, and that a change in the heme redox state or exogenous ligand binding to the heme iron complex relieves this suppression, as described above. These mutants were found to exist as tetramers and an analysis of several truncated mutants allowed mapping of the regions responsible for tetramer formation. It is presumed that the PAS-B domain in part contributes to the oligomer formation, as has been suggested for other PAS domains [8,9,10]. These studies revealed that only the tetramer is active toward c-AMP and suggested that oligomerization results in the formation of functional catalytic domain, implying that catalysis occurs via an inter-subunit mechanism. 

On the basis of molecular mass measured by multi-angle light scattering after gel filtration, it has also been proposed that the full-length *Ec* DOS protein is a dimer in aqueous solution, similar to many other heme-based gas-sensing enzymes [44]. It is not clear why this discrepancy in experimentally determined oligomeric state (dimer *vs*. tetramer) between gel exclusion chromatography and multi-angle light scattering exists. It is possible that the lengths of the proteins overexpressed in *E. coli* were different in the two sets of experiments. However, it should be noted that such discrepancies in oligomeric status determined using different methods are not uncommon for some eukaryotic proteins. For example, the oligomeric status of heme-regulated eukaryotic initiation factor 2α kinase (also known as heme-regulated inhibitor [HRI]) varies according to the methods used, although the same light-scattering technique that indicated *Ec* Dos was a dimer showed that HRI was a hexamer [45]. 

Oligomerization state may not be fully assigned using gel filtration unless analytical ultracentrifugation data has been carried out. Multi-angle light scattering measurement would exhibit clearly this limitation.

## 4. Physicochemical Characterizations

### 4.1. The K_d_ Values for O_2_ and CO Binding to Ec DOS are Very High

Because PDE activity toward c-di-GMP is substantially enhanced by binding of O_2_, it is reasonable to infer that O_2_ is the natural exogenous axial ligand for the heme Fe(II) complex of *Ec* DOS [11,12,24,25]. O_2_ ought to be a better H-bond acceptor than NO or CO as it is probably ferric-superoxide so it would interact more strongly with R97. A characteristic feature of *Ec* DOS is its very high *K*_d_ value (340 μM for the full-length *Ec* DOS; 20 μM for *Ec* DOS-PAS-A) for O_2_ binding, indicating that the affinity of *Ec* DOS for O_2_ is very low (Table 1) [46]. The *K*_d_ value of O_2_ for full-length *Ec* DOS was evaluated to be 74 μM by another group [11] (the *K*_d_ value was obtained by directly mixing the heme Fe(II) protein with 1.0–1,280 μM O_2_ dissolved in buffer and subsequently by evaluation from resultant spectral changes in [11]. On the other hand, the value was obtained by calculation from the *k*_on_ and *k*_off_ values for O_2_ binding in [46]). These high *K*_d_ values are comparable to that of FixL, another heme-based oxygen-sensor enzyme. Thus, it has been suggested that both *Ec* DOS and FixL act to detect changes in the relative O_2_ concentration under aerobic conditions. In contrast, the *K*_d_ values of globin-coupled oxygen sensors, another class of heme-based oxygen-sensor enzymes that contain a heme-bound globin fold, are much lower than those of *Ec* DOS and FixL, and are comparable to those of Hb and Mb [19,47,48]. This suggests that globin-coupled oxygen-sensor enzymes sense very low concentrations of O_2_ and, thus, play a role in regulating catalytic functions under (semi)-anaerobic conditions. 

**Table 1:** Reduction potential values, O_2_-binding rate constants (*k*_on_), O_2_-dissociation equilibrium constants (*K*_d_), and autoxidation rate constants (*k*_ox_) of *Ec* DOS-PAS-A WT (wild type) and mutant proteins.

	Reduction potential	*k*_on_	*K*_d_	*k*_ox_	Ref.
	(mV *vs*. SHE)	(×10^−3^ μM^−1^s^−1^)	(μM)	(min^−1^)	
WT	45–70	37–81	20–21	0.0053	[22,33,46,49]
D40A	95			0.051	[41]
D40N	114			0.033	
H83A				0.01	[42]
N84A				0.0015	
R85A				0.0026	
E86A				0.0054	
K89A				0.0043	
R91A				0.54	
E93A				0.0057	
S96A				0.0063	
M95A	−26	>1,000	<0.73	0.0013	[46,50]
M95H	−122	>1,000	<0.79	0.016	
M95I		160	1.4		[51]
M95L	−1	>1,000	<0.45	0.0017	[46,50]
R97A	43	76		>9.5	[33]
R97E	40			>45	
R97I	49	155	500	0.16	
L99T	23	49		0.049	[49]
L99F	24	75		0.37	
F113L	29	>200		0.00068	[42,52]
F113T	43	78		0.018	
F113Y	−27	26		0.039	
L115T	35	55		0.065	[49]
L115F				0.33	

However, it is not possible to make direct correlation of oxygen affinities and catalytic responses. Dose-response measurements must be done and they were done for FixL, which showed a sharp response in microaerobic oxygen concentration despite its low oxygen affinity. This has been explained by a “memory effect” (hysteresis) and showed to be in agreement to *in vivo* measurements [53]. For *Ec* DOS, it was also shown that there is no linear relationship between oxygen affinity and catalytic activity, where a sigmoidal response was observed [11]. It can be suggested that *Ec* DOS responds to a much more moderate oxygen drop than FixL and it would sense changes under aerobic conditions. A recent report showed that FixL with low O_2_ affinity (*K*_d_ ~700 μM) still exhibits very efficient oxygen response [54].

Although CO may not be the natural effector gas for *Ec* DOS, studies of CO-binding kinetics have provided valuable information on the electrostatic and structural properties of the distal heme environment of the heme protein. The *K*_d_ values of full-length *Ec* DOS and *Ec* DOS-PAS-A for CO are 3.1 and 0.6 μM, respectively. These values are also significantly higher than those of Hb and Mb [46].

### 4.2. O_2_ and CO Binding Kinetics and the Stability of the Heme Fe(II)-O_2_ Complex are Substantially Altered by Mutations at M95 and R97

M95 is the axial ligand on the heme distal side of the heme Fe(II) complex of *Ec* DOS (Figure 6, Figure 8) [27,28]. M95A and M95L mutations of *Ec* DOS-PAS-A-heme Fe(II) substantially altered the *k*_on_ value for O_2_ binding (>30-fold), whereas the same procedure does not significantly change the *k*_off_ value [46] (Table 1). As a consequence, the *K*_d_ values of M95 mutants for O_2 _binding are much lower than that of the WT, suggesting that the affinity of *Ec* DOS for O_2_ is substantially increased by M95 mutations [46]. A similar trend for CO binding to M95 mutants was also observed. Mutations of M95 to A or L result in a 5‑coordinated heme, opening the pocket on the heme distal side and allowing direct binding of exogenous ligands without prior dissociation of an endogenous ligand.

The autoxidation rate of the heme iron complex in heme proteins reflects the polar properties in the heme environment [55,56]. Autoxidation rate constants are three-fold lower than that of WT for *Ec* DOS-PAS-A M95A and M95L mutants (Table 1), which change the polar characteristics of the heme Fe(II)-O_2_ complex environment, including the water molecule in the heme distal site [55,56]. M95 should also play some role in destabilizing the heme Fe(II)-O_2_ complex in *Ec* DOS. 

R97 is the direct O_2_-binding site in the heme Fe(II)-O_2_ complex [28] (Figure 8). Thus, it is not surprising that R97 mutants exhibit substantially altered O_2_-binding kinetics. Notably, *k*_on_ rates of R97 mutants are several-fold higher than that of the WT, and autoxidation rate constants of R97A and R97E are more than 400-fold higher than that of the WT [33] (Table 1). These observations indicate that the R97 residue is important for the stability of the heme Fe(II)-O_2_ complex.

### 4.3. Cyanide and Imidazole Binding is Influenced by Mutations at M95 and R97

The cyanide anion (CN^−^) is not a natural exogenous axial ligand for the heme protein. However, analyses of CN^−^ binding to the heme Fe(III) complex of *Ec* DOS have provided valuable information on the character of the heme distal environment [57,58]. The *k*_on_ values of CN^−^ for M95A and M95L in full-length *Ec* DOS were 600-fold higher than that of the WT enzyme, and that for F113L was 170-fold higher [26]. The *k*_on_ values of R97A, R97E, and R97I, for CN^−^ were several-fold lower than that of WT for both full-length *Ec* DOS and *Ec* DOS-PAS-A. 

Similar kinetic analyses have been conducted for binding of *Ec* DOS and imidazole, which, like CN^−^ is not a natural exogenous axial ligand for *Ec* DOS [26]. The *k*_on_ values of M95A and F113L mutants for binding of imidazole to *Ec* DOS-heme Fe(III) were 11-fold and 130-fold higher, respectively, than that of the WT. The *k*_on_, *k*_off_, and *K*_d_ values of other M95, R97, and F113 mutants were also significantly different from those of the WT protein. 

### 4.4. Reduction Potential Values are Important for O_2_-Regulated Catalysis

Reduction potential values of the heme protein reflect the equilibrium between heme Fe(III) and Fe(II) complexes or, in some cases, the stability of the heme Fe(II)-O_2_ complex; both are significantly influenced by the electrostatic environment of the heme iron complex [55,56]. The reduction potential value of *Ec* DOS-PAS-A WT obtained at pH 7.0 was approximately 70 mV *versus* the standard hydrogen electrode (SHE; Table 1) [22,33,46,49], suggesting that the heme Fe(II) complex in *Ec* DOS-PAS-A is thermodynamically more stable than the heme Fe(III) complex in aqueous solution. It is reasonable that *Ec* DOS would adopt a heme Fe(II) form *in vivo* under aerobic conditions because this form is prepared to accept the O_2_ molecule, allowing subsequent stimulation of catalysis in *E. coli* cells. Interestingly, mutations at M95 substantially decreased the reduction potential value to −122 mV *versus* SHE (Table 1) [46,50]. The coordination of M95 to the heme Fe(II) complex would be important in maintaining an optimal, positive reduction potential as well as locking catalysis in the “off” state, until relieved by O_2_ binding. Mutations at D40 on the heme proximal side have been shown to increase reduction potential [41], whereas those on the heme distal side and the F-G loop, with the exception of F113Y, do not substantially change it (Table 1) [42,49,52], suggesting that the structure and/or electrostatic characteristics of the heme proximal side rather than those of the heme distal side are important for maintaining a heme reduction potential that is optimal for O_2_-regulated catalysis by *Ec* DOS. 

### 4.5. Infrared Spectra are Not Changed by Mutations at M95

The infrared spectrum reflects the vibration of specific sets of chemical bonds within a molecule and can be used as a fingerprint for the identification of unknown compounds. The infrared spectrum of the heme Fe(II)-CO complex provides useful information on the CO environment of this complex in the heme protein. Except for M95A, the C-O stretching modes in the heme Fe(II)-CO complexes of *Ec* DOS-PAS-A M95 mutants showed no difference in band positions from those of the WT protein [46]. This finding is surprising, given that *K_d_* and *k_on_* values for the association of CO with *Ec* DOS-PAS-A are substantially affected by mutations at M95. 

### 4.6. Resonance Raman Spectra: Role of Hydrogen-Bonding Networks Involving M95, R97, W53, Y126, Heme Propionate, and Heme Vinyl in Signal Transduction

Resonance Raman (RR) spectra are very useful tools for understanding the structure and ionic characteristics of the heme environment of heme proteins [2]. Notably, the protein structure near aromatic amino acid residues in the protein can be elucidated using ultraviolet RR spectra. RR spectroscopy has been successfully used to address structure-function relationships of *Ec* DOS, particularly with respect to intramolecular signal transduction.

The RR spectra of heme Fe(III) and heme Fe(II) complexes of *Ec* DOS-PAS-A were characteristic of 6-coordinated low-spin complexes [59,60]. The RR spectrum of the heme Fe(II)-CO complex revealed a nearly linear Fe-C-O geometry with an upright structure and a fairly hydrophobic distal side pocket in the heme environment. On the basis of the RR spectra of the heme Fe(II) complex of H77 and M95 mutants, it was suggested that H77 and M95 are the axial ligands for the heme Fe(II) complex of *Ec* DOS-PAS-A. Picosecond time-resolved RR spectra of the photo-dissociated heme Fe(II)-CO complex of the WT protein exhibits an Fe-His stretching band (ν_Fe-His_) at 214 cm^−1^. In contrast, the photodissociated heme Fe(II)-CO complex of the H77A mutant does not exhibit this ν_Fe-His_ band, but does yield a ν_Fe-Im_ band in the presence of exogenous imidazole. These results suggest that exogenous imidazole is inserted between the heme Fe(II) complex and the protein surface of the H77A mutant.

As mentioned above, M95 is the axial ligand for the heme Fe(II) complex and forms a hydrogen bond with the heme 7-propionate [27,28]. Upon O_2_ binding to the heme Fe(II) complex, M95 is replaced by O_2_, and heme 7-propionate forms a hydrogen bond with R97 instead of M95 [28]. Thus, replacement of the distal axial ligand from M95 with O_2_ influences the heme 7-propionate hydrogen-bond network, resulting in large conformational changes in the F-G loop and stimulation of catalysis. R97 also serves as the O_2_-binding site in the heme Fe(II)-O_2_ complex [28]. RR results revealed significant interactions of R97 and F113 with a ligand bound to the 6th coordination site of the heme Fe(II) complex and profound structural changes in the heme propionates upon dissociation of the ligand. These observations suggest that the electrostatic interaction of R97 with the heme 7-propionate, and steric interaction of F113 with the ligand are crucial for regulating the competitive recombination of M95 and the ligand to the heme Fe(II) complex [61].

Ultraviolet RR spectra show that the environment surrounding W53, which contacts the 2-vinyl group of heme, is changed to a more hydrophobic environment upon O_2_ binding, whereas it is changed to a more hydrophilic environment by CO binding. The binding of O_2_ or CO to the heme produces drastic changes in the Y126-bound I_β_-strand at the protein surface. Specifically, it was found that N84 forms a hydrogen bond with Y126 in either the O_2_- or CO-bound heme Fe(II) complex, but not in the ligand-free heme Fe(II) complex. These observations suggest that the hydrogen-bonding network from the heme 6-propionate to Y126 through N84 is important in transducing the signal from the heme-binding PAS domain to the catalytic domain in *Ec* DOS [62]. 

Ultraviolet RR spectra also showed that W53, Y126, and Y80 are located near the 2-vinyl, 4-vinyl, and propionate side chains of heme, respectively [63]. On the basis of these observations, it was suggested that heme redox changes induce structural changes of the heme side chains, which are then communicated to the protein surface of the PAS sensor domain through these amino acids, resulting in global changes in protein conformation that should be associated with the redox-dependent catalytic simulation of *Ec* DOS. 

Time-resolved ultraviolet RR spectral analyses of Raman bands of W54, which is located near the heme 2-vinyl side chain, have revealed that rapid (<0.5 ns) protein conformational changes occur upon CO photodissociation, with nanosecond structural relaxation, followed by CO geminate recombination [64]. These studies also showed that heme structural changes, particularly those of vinyl and propionate side chains, trigger conformational changes of the protein matrix on a nanosecond to microsecond time scale. 

At initial stages in the study of *Ec* DOS, RR spectra of WT and mutant proteins were reported [51,60]. However, spin states, optical absorption, and electron paramagnetic resonance (EPR) spectra reported in these studies are significantly different from those published later by other groups, resulting in differences in the coordination structures of WT and mutant proteins. It is likely that sample solutions used in these earlier reports contained significant amounts of denatured forms of these proteins, resulting in spectral differences from those of the native proteins, and thus incorrect assignments. 

### 4.7. Pulse Radiolysis: Allosterically Regulated O_2_ Binding

Pulse radiolysis is a powerful tool for investigating electron transfer within proteins, often allowing an electron to be introduced rapidly and selectively into one redox center of an enzyme [65]. Binding of the O_2_ ligand following reduction of the heme iron complex in dimeric *Ec* DOS-PAS-A-heme Fe(III) was examined using pulse radiolysis [66]. The kinetics of O_2_ ligation to the heme Fe(II) complex in the dimer was a two-phase process reflecting the stepwise reduction of two heme complexes upon application of a high-dose pulse. It was proposed that the faster phase corresponds to binding the first O_2_ molecule to one subunit of the dimer, followed by binding of the second O_2_ molecule to the other subunit. Notably, for the heme axial ligand mutant proteins, M95A and M95L, O_2_ binding displayed single-phase kinetics [66]. 

### 4.8. Ultrafast Ligand Rebinding: Allosterically Regulated Catalysis and Involvement of Met95 in Signal Transduction

The properties of CO or O_2_ binding to the heme in dimeric and monomeric *Ec* DOS-PAS-A, occurring on a microsecond to second time scale, were compared with those of full-length *Ec* DOS [44]. The ligand kinetics were found to be influenced by (i) the presence of the catalytic domain, (ii) dimerization state, and (iii) the ligation state of the other subunit. These results, taken together with steady-state titrations, imply allosteric interactions within dimers. An analysis of a variety of time-resolved experiments on a picosecond scale showed that M95 plays a major role in intradimer interactions [44,67]. Ligand-induced M95 rearrangements (dissociation of M95 from the heme Fe(II) complex, followed by ligand binding) are predicted to occur along the signaling pathway involving the flexible F-G loop and have been suggested to be important in transducing the signal that changes the activity of the catalytic domain [37,44,67,68,69]. 

## 5. Site-Directed Mutations at Sites other than M95 and R97

### 5.1. Mutations at L99, F113, and L115 on the Heme Distal Side Change Autoxidation Rate Constants and Reduction Potentials

The X-ray crystal structures of *Ec* DOS-PAS-A showed that L99, F113, and L115, indirectly and directly, form a hydrophobic triad on the heme distal side located at or near the ligand access channel of the heme iron [27,28]. In general, mutations at L99 and L115 increased the autoxidation rate constant and decreased the reduction potential value [49], with the corresponding Phe mutants exhibiting the largest increase in autoxidation rate constant. Conversion of F113 to Y decreased the reduction potential value, whereas conversion of F113 to L markedly increased the *k*_on_ value for O_2_ [52,70] (Table 1). In sharp contrast to other point mutations in this hydrophobic triad, mutation of F113 to L substantially decreased the autoxidation rate constant. Collectively, these results suggest that these heme distal side hydrophobic residues are critically important in determining reduction potential value, ligand binding, and stability of the heme Fe(II)-O_2_ complex. 

### 5.2. Mutations at D40 at the Heme Proximal Side Abolish Catalytic Activity toward c-AMP and Change Autoxidation Rate Constants and Reduction Potential Values

The X-ray crystal structure showed that D40 is located near the F-G loop, forms a hydrogen bond with H77 of the proximal heme axial ligand via two water molecules, and forms a salt bridge with R85 at the protein surface [27,28]. D40 mutants lose activity toward c-AMP [41], suggesting that the F-G loop is significantly involved in *Ec* DOS intramolecular signal transduction. Autoxidation rate constants and reduction potential values of these mutants were significantly higher than those of the WT (Table 1) [41], suggesting that D40 plays a critical role in determining the electrostatic properties of the heme environment. 

### 5.3. Amino Acids in the F-G Loop are Crucial for Heme Affinity and Autoxidation Rate Constant (i.e., Stability of the Heme Fe(II)-O_2_ Complex)

Ionic and non-ionic polar amino acids in the F-helix and F-G loop (E86-R97) are predicted to play critical roles in intramolecular signal transduction from the heme-bound PAS domain to the PDE domain. This supposition was experimentally tested by generating eight Ala single mutants (H83A, N84A, R85A, E86A, K89A, R91A, E93A, and S96A) and a triple mutant (K89A/R91A/E93A) of full-length *Ec* DOS and examining their heme environments and catalytic activities toward c-di-GMP [42]. The mutants had low heme affinities and their autoxidation rate constants were significantly different from that of WT (Table 1), suggesting that these residues in the F-G loop form a heme distal architecture that confers stability to the heme Fe(II)-O_2_ complex. 

### 5.4. Fluorescence Spectra Suggest that W53 and W110 are Located Near the Heme and on the Protein Surface, Respectively

Fluorescence bands of W53F and W110I and their complexes with 8-aniline-1-naphthalenesulfonic acid were compared with those of WT [71]. Bands of W110I in *Ec* DOS-PAS-A were much weaker than those of WT, suggesting that the fluorescence of the remaining W53 residue is quenched by interactions with heme and that the W110 residue is exposed to the solvent [71]. These results are consistent with the protein structure [27,28] and RR spectra of *Ec* DOS-PAS-A [64].

## 6. Genetic Studies

The role of *Ec* DOS in *E. coli* was evaluated by examining the morphology, growth, and motility of *E. coli* cells lacking the *dos* gene. In the absence of the *dos* gene, *E. coli* cells were filamentous compared with WT cells, suggesting that *Ec* DOS is involved in cell division [72,73]. Consistent with this, the growth rate of the *dos*-knockout cells was also significantly lower than that of the WT cells under aerobic conditions [72]. These observations suggest that *Ec* DOS is involved in various important physiological functions of *E. coli* cells. 

The idea that c-AMP is a bone fide substrate of *Ec* DOS has been greeted with a certain degree of skepticism, as the PDE activity of *Ec* DOS toward c-AMP is very low and the amino acid sequence of the C-terminal domain predicts that c-di-GMP is the native substrate for *Ec* DOS [16,17,18,20,23]. However, the concentration of c-AMP in *dos*-knockout *E. coli* cells is reported to be 17-fold higher than that in WT cells [73]. Moreover, the addition of excess c-AMP to the medium of WT *E. coli* cells to adopt a filamentous morphology, similar to that observed for the *dos*-knockout cells [73]. These observations suggest that c-AMP is also a substrate of *Ec* DOS. It is also possible that c-di-GMP plays the similar role in the bacterial behavior. 

In order to understand how *Ec* DOS is involved in cell division, researchers evaluated differences in the transcriptional activity of 29 genes related to cell division between WT and *dos*-knockout cells using classical reverse transcription-polymerase chain reaction (RT-PCR) and real-time PCR. In addition, the role of c-AMP in physiological functions was assessed by applying microarray analyses to *E. coli* cells deficient in the gene encoding c-AMP receptor protein (CRP) [73]. This study revealed that 11 genes associated with cell division are regulated by the *dos* gene, of which four are associated with CRP. These results suggest that *Ec* DOS is significantly involved in regulating cell division and further implicate CRP in these transcriptional activities of *Ec* DOS.

However, there are limitations of the biological data where there was in part a lack in complement the knockout genes or the use of domesticated strains for proper biofilm formation.

## 7. Application of a Protein Microarray to Study *Ec* DOS Interactions

**Figure 10:**
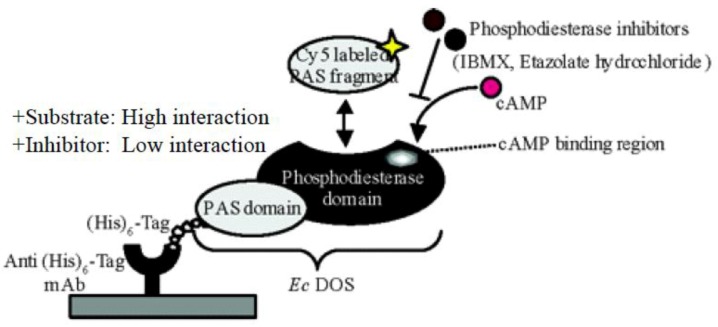
The protein microarray demonstrated that the catalytic activity of *Ec* DOS is closely associated with its protein–protein interactions [30,31]. Full-length (His)_6_-tagged *Ec* DOS was immobilized on a solid surface via an anti-(His)_6_ monoclonal antibody (mAb). This protein microassay showed that *Ec* DOS-PAS-A-heme Fe(II) interacted with full-length *Ec* DOS, whereas *Ec* DOS-PAS-A-heme Fe(III) or heme-free *Ec* DOS-PAS-A did not interact with the enzyme. Addition of c-AMP increased the interaction between labeled *Ec* DOS-PAS-A and full-length *Ec* DOS, whereas addition of inhibitors decreased this interaction. These findings correspond closely with the catalytic regulation of full-length *Ec* DOS by *Ec* DOS-PAS-A (Figure 7) [21]. Adapted from [31].

A protein microarray that is highly efficient in detecting protein–protein interactions was developed using *Ec* DOS. This assay makes use of exogenously expressed proteins containing an N- or C-terminal (His)_6_-tag, which facilitates efficient protein purification using affinity chromatography, and an anti-(His)_6_ antibody, which binds to (His)_6_-tagged proteins with high affinity, and is thus ideal for protein microchip applications [30,31]. In this application, anti-(His)_6_ antibodies are first immobilized on the plate, after which a solution containing overexpressed (His)_6_-tagged protein is applied. Once the protein is bound to the plate-attached antibody via its (His)_6_-tag, it can easily swing on the plate with more freedom and thus becomes more accessible to other proteins with which it might be capable of interacting (Figure 10). 

This protein microarray revealed that *Ec* DOS-PAS-A-heme Fe(II) interacts with full-length *Ec* DOS-heme Fe(II) with high affinity, whereas *Ec* DOS-PAS-A-heme Fe(III) and heme-free *Ec* DOS-PAS-A do not interact with full-length *Ec* DOS-heme Fe(II). These findings are in good agreement with the results obtained from assays of *Ec* DOS catalytic activity toward c-AMP, as described in Figure 7 [21]. Addition of the substrate c-AMP enhanced the interaction, whereas addition of inhibitors, such as etazolate hydrochloride and 3-isobutyl-1-meth-ylxanthine (IBMX), decreased protein–protein interaction. Site-directed mutants (at H590 and H594) containing substitutions at the catalytic site of full-length *Ec* DOS did not interact with *Ec* DOS-PAS-A-heme Fe(II). Thus, the novel protein microarray system described here employing the (His)_6_-tag, which is commonly used for protein engineering, has potentially wide applicability in the study of protein–protein interactions and the identification of new proteins that interact with a protein of interest. 

Here, it is important to mention that (His)_6_-tag insertion can cause severe problems in some proteins even leading to the lack of activity. Note that etazolate hydrochloride is a selective inhibitor for c-AMP-specific PDEs, whereas 3-isobutyl-1-meth-ylxanthine is a nonspecific inhibitor for both c-AMP and c-GMP PDEs [22]. Both inhibitors strongly inhibited the c-AMP PDE activity of *Ec* DOS and hampered the protein–protein interaction in terms of the protein microarray. However, the binding site and binding fashion of c-AMP and those inhibitors in the *Ec* DOS protein are not known.

## 8. Conclusions

The heme-based oxygen-sensor PDE, *Ec* DOS, has been efficiently overexpressed in *E. coli*, purified, and shown to be stable [13,14]. Thus, its biochemical and physicochemical properties applications have been extensively studied as a prototypical heme-based oxygen-sensor enzyme. 

It should be noted that the heme-free form of *Ec* DOS, like that of other heme-based oxygen-sensor enzymes such as YddV and *Af*GcHK [19,47], has ample catalytic activity toward c-di-GMP [26]. Thus, the purpose of the heme iron complex in the protein is to suppress catalysis; this catalytic suppression is relieved by binding of an exogenous ligand, such as O_2_, to the heme Fe(II) complex in the oxygen-sensor PAS domain of *Ec* DOS [25]. Although *Ec* DOS clearly acts as an oxygen-sensor PDE toward c-di-GMP, its catalytic activity is also simulated by binding of other exogenous gas molecules, such as CO and NO, to the heme Fe(II) complex, and by binding of exogenous chemicals, such as cyanide and imidazole, to the heme Fe(III) complex [11,12,24,25,26]. Upward movement or twisting of amino acid residues on the heme distal side, caused by the binding of exogenous axial ligand or mutations at M95 (the axial ligand to the heme Fe(II) complex), triggers intramolecular signal transduction, switching on catalysis in the C-terminal domain. *Ec* DOS may work chemically toward c-AMP as the redox sensor, but its physiological role as the redox sensor is unlikely. It has been accepted in general that *Ec* DOS is an oxygen sensor toward c-di-GMP in *E. coli*.

## Abbreviations

c-di-GMP: cyclic diguanylate monophosphate, bis(3'-5')-cyclic dimeric guanosine monophosphate
CRP: c-AMP receptor protein; c-AMP-dependent transcriptional regulator
DGC: diguanylate cyclase; synthesis of c-di-GMP
*Ec* DOS: *E. coli* Direct Oxygen Sensor; heme-based oxygen-sensor phosphodiesterase from *E. coli*, also designated *Ec* DosP
*Ec* DOS-heme Fe(II): full-length *Ec* DOS containing the heme Fe(II) complex
*Ec* DOS-heme Fe(III): full-length *Ec* DOS containing the heme Fe(III) complex
*Ec* DOS-PAS-A: isolated heme-bound N-terminal domain containing a PAS structure
*Ec* DOS-PAS-A-heme Fe(II): *Ec* DOS-PAS-A containing the heme Fe(II) complex
*Ec* DOS-PAS-A-heme Fe(III): *Ec* DOS-PAS-A containing the heme Fe(III) complex
*Ec* DosC: heme-based oxygen sensor diguanylate cyclase from *E. coli*, also designated YddV
*Ec* DosP: heme-based oxygen-sensor phosphodiesterase from *E. coli*, also designated *Ec* DOS
Hb: Hemoglobin
heme Fe(II): protoporphyrin IX-Fe(II) complex
heme Fe(III): protoporhyrin IX-Fe(III) complex, or hemin
*K*_d_: equilibrium dissociation constant
*k*_off_: dissociation rate constant
*k*_on_: association rate constant
l-di-GMP: linear diguanylate monophosphate, pGpG
Mb: Myoglobin
PAS: an acronym formed from Per (*Drosophila* period clock protein)- Arnt (vertebrate aryl hydrocarbon receptor nuclear translocator)- Sim (*Drosophila* single-minded protein)
PDE: phosphodiesterase; linearization of c-di-GMP
RR spectroscopy: resonance Raman spectroscopy
WT: wild type
YddV: heme-based oxygen sensor diguanylate cyclase from *E. coli*, also designated *Ec* DosC
